# Expression of *SEC62* Oncogene in Benign, Malignant and Borderline Melanocytic Tumors—Unmasking the Wolf in Sheep’s Clothing?

**DOI:** 10.3390/cancers13071645

**Published:** 2021-04-01

**Authors:** Cornelia S. L. Müller, Claudia Pföhler, Maria Wahl, Florian Bochen, Sandrina Körner, Jan Philipp Kühn, Alessandro Bozzato, Bernhard Schick, Maximilian Linxweiler

**Affiliations:** 1 Department of Dermatology, Venerology and Allergology, Saarland University Medical Center, D-66421 Homburg, Germany; cornelia.mueller@uks.eu (C.S.L.M.); claudia.pfoehler@uks.eu (C.P.); mariawahl@aol.com (M.W.); 2 Department of Otorhinolaryngology, Head and Neck Surgery, Saarland University Medical Center, D-66421 Homburg, Germany; florian.bochen@uks.eu (F.B.); sandrina.koerner@uks.eu (S.K.); jan.kuehn@uks.eu (J.P.K.); alessandro.bozzato@uks.eu (A.B.); bernhard.schick@uks.eu (B.S.)

**Keywords:** melanoma, SEC62, carcinogenesis, prognostic biomarker, metastasis

## Abstract

**Simple Summary:**

Amplification and overexpression of the *SEC62* oncogene was reported in a variety of human cancers and was associated with poor prognosis as well as lymph node and distant metastases. In this study, *SEC62* expression was analyzed in benign, borderline, and malignant melanocytic lesions of 209 patients. We found the highest expression in Spitz nevi, followed by melanoma metastases, primary melanoma, congenital nevi, and blue nevi. In melanoma patients, high Sec62 levels correlated with shorter overall and progression-free survival. Significantly higher Sec62 levels were found in melanomas with lymph node and distant metastases compared with those without. Taken together, these data suggest a relevant role of *SEC62* as a metastasis-stimulating oncogene in melanoma development, which represents a promising therapeutic target.

**Abstract:**

*SEC62* oncogene located at chromosomal region 3q26 encodes for a transmembrane protein of the endoplasmic reticulum (ER) and is expressed at high levels in numerous human malignancies. *SEC62* overexpression has been associated with worse prognosis and high risk for lymphatic and distant metastases in head and neck cancer, cervical cancer, hepatocellular cancer, and lung cancer. However, its role in the development and tumor biology of melanocytic lesions has not been investigated so far. An immunohistochemical study including 209 patients with melanocytic lesions (malignant melanoma (MM), *n* = 93; melanoma metastases (MET), *n* = 28; Spitz nevi (SN), *n* = 29; blue nevi (BN), *n* = 21; congenital nevi (CN), *n* = 38) was conducted and *SEC62* expression was correlated with clinical data including patient survival and histopathological characteristics. SN showed the highest *SEC62* expression levels followed by MET, MM, CN, and BN. High *SEC62* expression correlated with a shorter overall and progression-free survival in MM patients. Additionally, high Sec62 levels correlated significantly with higher tumor size (T stage), the presence of tumor ulceration, and the presence of lymph node as well as distant metastases. Strikingly, *SEC62* expression showed a strong correlation with Clark level. Taken together, these data demonstrate that *SEC62* is a promising prognostic marker in MM and has the potential to predict biological behavior and clinical aggressiveness of melanocytic lesions.

## 1. Introduction

The *SEC62* gene located at the long arm of chromosome 3 (3q26.2) encodes for a transmembrane protein of the endoplasmic reticulum (ER) and is a component of the human Sec complex, which is responsible for the translocation of precursor proteins from the cytosol into the endoplasmic reticulum (ER) lumen, as well as the regulation of intracellular calcium homeostasis. This translocation complex consists of a distinct subset of proteins, three of which belong to the Sec group of proteins: *Sec61*, Sec62, and Sec63 [1,2]. Sec62, as the smallest member of this group with a molecular weight of 45.9 kDa, consists of two transmembrane helical domains with a cytoplasmic N- and C-terminus. Recent data suggest that in eukaryotic cells, Sec62 is involved in the post-translational transport of short secretory and transmembrane precursor proteins, which is mediated through a direct interaction of Sec62 with Sec61 and the ribosome [3,4,5]. Apart from this, Sec62 harbors two putative cytosolic EF hand domains and is able to inhibit the passive calcium efflux through the Sec61 channel into the cytosol possibly mediated through calcium-dependent conformational changes in the Sec complex [6,7,8,9]. Amplification of the 3q26 region and overexpression of the *SEC62* gene have been found in several cancer entities over the past decades including head and neck squamous cell carcinomas (HNSCC) [10,11], prostate cancer [12], esophageal cancer [13], cervical cancer [14,15], ovarian cancer [16], breast cancer [17], and non-small cell lung cancer (NSCLC) [18]. For non-small cell lung cancer, head and neck squamous cell carcinomas, and breast cancer, high *SEC62* expression levels are a strong predictor of poor clinical outcome and correlate significantly with the presence of lymph node and/or distant metastases [17,19,20,21,22]. In hepatocellular cancer, high *SEC62* expression is associated with a higher risk of recurrence after surgical treatment [23]. Beneath its potential role as a prognostic biomarker in the aforementioned cancer entities, Sec62 directly influences tumor cell biology by stimulating cancer cell migration and invasion, as well as enabling tumor cells to recover from ER stress through a molecular mechanism called recovER-phagy [8,24,25,26,27,28]. These effects can explain how tumor cells profit from an increased *SEC62* expression level and might be responsible for the poor prognosis of *SEC62*-overexpressing tumors. Based on the finding that the stimulation of cancer cell migration by Sec62 is probably mediated through its influence on the calcium efflux from the ER lumen, the calmodulin inhibitor trifluoperazine (TFP) was identified as a potent agent to antagonize the calcium effect of Sec62 on cellular calcium level, and thereby counteracts Sec62 mediated cancer cell migration and metastasis [8]. Hence, TFP represents a promising small molecule for antimetastatic therapy in *SEC62*-overexpressing tumors whose therapeutic potency can be stimulated by adding the SERCA inhibitor thapsigargin (TG) [8,29]. However, the exact molecular mechanisms of how Sec62 can influence tumor cell migration and invasion through a regulation of cytosolic calcium levels remain elusive.

In the field of dermatological oncology, only one study addressed *SEC62* expression and its role in dermatological carcinogenesis so far [30]. Müller et al. investigated Sec62 protein levels in 41 atypical fibroxanthomas (AFX) and found markedly increased *SEC62* expression in lesional tissue compared with the adjacent healthy squamous epithelium. Sec62 levels were higher in AFX with tumor necrosis, tumors with advanced Clark levels, and tumors with a size >5 cm^2^. The expression of the *SEC62* gene in benign, malignant, and dysplastic melanocytic tumors, as well as its predictive and prognostic value, have not yet been investigated by any other study. 

To date, only a few features in primary melanoma exist that represent clinically relevant prognostic markers. Tumor thickness and Breslow depth are the most accurate prognostic markers for patient survival in early-stage cutaneous melanoma. The mitotic rate and Ki-67 expression, both representing markers of proliferation, are also significant predictors of patient survival. Expressions of melanoma cell adhesion molecule (MCAM) and metallothionein I and II were shown to be independent prognostic markers of prognosis in primary melanoma [31]. However, the prognostic and predictive validity of the aforementioned biomarkers has been differentially valued by several studies, resulting in an ongoing debate regarding which markers have enough evidence to guide clinical decisions. Though several potential biomarkers have intensively been investigated in retrospective as well as prospective studies [32], only ulceration and Breslow thickness are incorporated in the new eighth edition of the AJCC staging system [33]. In this regard, more reliable and clinically relevant markers are urgently needed for better therapy management and outcome prediction.

We therefore investigated the expression of 3q-encoded oncogene *SEC62* in a total of 209 benign, malignant, and borderline melanocytic tumors and correlated *SEC62* expression level with the patients’ clinical data and histopathological characteristics.

## 2. Materials and Methods

### 2.1. Patient Characteristics and Tissue Samples

All formalin-fixed, paraffin-embedded tissue samples (FFPE) from benign, borderline, and malignant melanocytic lesions that had been surgically removed between 2006 and 2016 were retrieved from the histopathology archives of the Department of Dermatology of the Saarland University Medical Center (Homburg, Germany). Samples from 209 patients were investigated in this study in total. The following clinical and histopathologic characteristics were recorded: sex, age, site, and laterality of lesion, as well as TNM stages for melanoma cases. 

During the sampling period, three different editions of the TNM classification existed. To ensure uniformity in the assessment of clinical stages, we used the AJCC classification 2009 throughout as this staging system covers the major period of time when patients were included. Clinical information was obtained from the hospital medical records of the referring clinicians. Histopathological diagnoses of the included cases comprised malignant melanoma (MM; *n* = 93), metastases of MM (MET; *n* = 28), Spitz nevi (SN; *n* = 29), blue nevi (BN; *n* = 21), and congenital nevi (CN; *n* = 38). In total, 109 men and 100 women were included with mean ages of 55.9 and 47.7 years, respectively. All available details of clinical and histopathological characteristics of the melanoma patients are summarized in Table 1. Among the 93 MM patients, 16 showed distant metastases with localizations in the lung (*n* = 14), liver (*n* = 5), brain (*n* = 2), bone (*n* = 2), and adrenal gland (*n* = 2). Out of the 16 MM patients with distant metastases, *n* = 9 had tumor size (T stage) 4, *n* = 5 had T stage 3, and *n* = 2 had T stage 2. Melanoma subtype of these patients was NMM for *n* = 13 patients, ALM for *n* = 2 patients, and SMM for *n* = 1 patient. All investigations were performed after obtaining approval by a local Human Investigations Committee of the Saarland Medicines Agency Ethics Review Board (approval number 281/10). All investigations presented herein were performed in accordance with established ethical standards and the Declaration of Helsinki. All patients gave their informed consent for scientific use of their tissue and respective clinical data.

### 2.2. Immunohistochemical Analysis

FFPE tissue sections were used for immunohistochemical staining targeting the Sec62 protein. After omitting the first three 10 µm sections of each FFPE block, consecutive 4 µm sections were prepared using a Leica RM 2235 rotary microtome (Leica Microsystems, Wetzlar, Germany), transferred onto Superfrost Ultra Plus microscope slides (Menzel-Gläser, Braunschweig, Germany) and dried in an incubator at 37 °C for at least 4 h. Upon deparaffinization, heat-induced epitope retrieval was performed by incubating the prepared slides in 10 mM citrate buffer (pH 6.0) at 95 °C for 30 min. In the next step, unspecific protein binding sites were blocked with 3% bovine serum albumin (BSA) (Sigma Aldrich, St. Louis, MO, USA) in phosphate-buffered saline (PBS) for 30 min at room temperature. Subsequently, the slides were incubated with the primary antibody using an affinity-purified polyclonal rabbit antipeptide antibody directed against the C terminus of human Sec62 (self-made). For each staining series, a specimen taken from a subcutaneously grown tumor in mice after local injection of UM-SCC1 cells (*SEC62* overexpressing and HPV16-negative human head and neck squamous cell carcinoma cell line) was used as the positive control, while negative controls were made by omitting the primary antibody in the staining protocol. Thereafter, visualization was performed using the REAL^TM^ detection system Alkaline Phosphatase (Dako Agilent Technologies, Glostrup, Denmark), according to the manufacturer’s instructions, and the slides were counterstained with hematoxylin (Dako Agilent Technologies, Glostrup, Denmark). Sec62 immunoreactivity was evaluated using the well-established immunoreactive score (IRS) according to the publication by Remmele and Stegner [34] with values ranging from 0 to 12 (0–1: no expression, 2–3: mild expression, 4–8: moderate expression, 9–12: strong expression). For IRS calculation, the intensity of staining (0: no reaction, 1: mild reaction, 2: moderate reaction, 3: intense reaction) as well as the percentage of stained lesional cells (0: no positive cells, 1: <10% of positive cells, 2: 10–50% positive cells, 3: 51–80% positive cells, 4: >80% positive cells) were multiplied to represent the final IRS. Immunohistochemical stainings were evaluated by three experienced examiners including one dermatopathologist and two postdoctoral research fellows. Mean values of the three scorings were used for statistical analysis. In total, 209 cases were available for immunohistochemistry with Sec62.

### 2.3. Statistical Analysis

GraphPad Prism 7.0 (GraphPad Software, La Jolla, CA, USA) and SPSS version 16 (IBM, Ehningen, Germany) were used for statistical analysis, presuming a significance level of 5% (α = 0.05) and a statistical power of 80% (β = 0.8). In testing the significance of thresholds, the existence of normal distribution was controlled by the Kolmogorov–Smirnov test and homogenous variance was checked by the Levine test. If parameters showed no normal distribution in the aforementioned tests, nonparametric Mann–Whitney U-test was used. In case of normal distribution, a two-sided *t*-test was performed. For multiple comparisons, a one-way ANOVA test was used. In the figures, statistical significance levels are indicated with stars (n.s.—non significant; *—*p* < 0.05; **—*p* < 0.005; ***—*p* < 0.001). All *p*-values < 0.05 were considered statistically significant. 

## 3. Results

### 3.1. Impact of *SEC62* Expression Level on the Prognosis of Melanoma Patients

**Figure 1:**
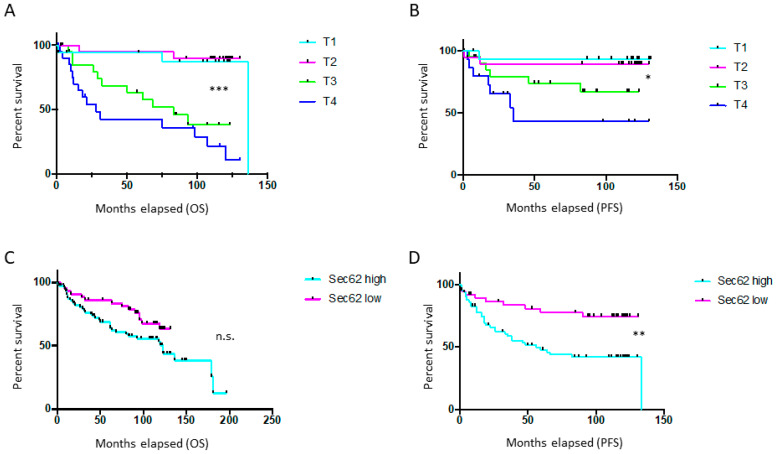
Survival of melanoma patients depending on T stages (malignant melanoma (MM) only) and *SEC62* expression level (MM + melanoma metastases (MET)): (**A**) overall survival (OS) T1 vs. T2 vs. T3 vs. T4l (**B**) progression-free survival (PFS) T1 vs. T2 vs. T3 vs. T4; (**C**) OS Sec62-high (*n* = 75) vs. Sec62-low (*n* = 46); (**D**) PFS Sec62-high vs. Sec62-low; log-rank test. Median OS was 80.41 months and median PFS was 57 months for all MM patients; n.s.—non significant; *—*p* < 0.05; **—*p* < 0.005; ***—*p* < 0.001.

First, overall survival (OS) and progression-free survival (PFS) were compared for melanoma patients with high (IRS 8–12) vs. low (IRS 0–7) *SEC62* expression and for patients with different T stages. As expected, OS and PFS improved for patients with T1 and T2 MM compared with patients with T3 and T4 melanoma (*p* < 0.0001 for OS, *p* = 0.008 for PFS, log-rank test; Figure 1A,B). For *SEC62*, high expression correlated with a shorter OS (*p* = 0.08; Figure 1C) and a significantly shorter PFS (*p* = 0.003; Figure 1D). The median OS was 78.41 months for Sec62-high MM patients compared with 83.63 months for Sec62-low MM patients. Median PFS was 47.68 months for Sec62-high MM patients compared with 71.11 months for Sec62-low MM patients. 

### 3.2. Correlation of SEC62 Expression with Clinical and Histopathological Features

**Figure 2:**
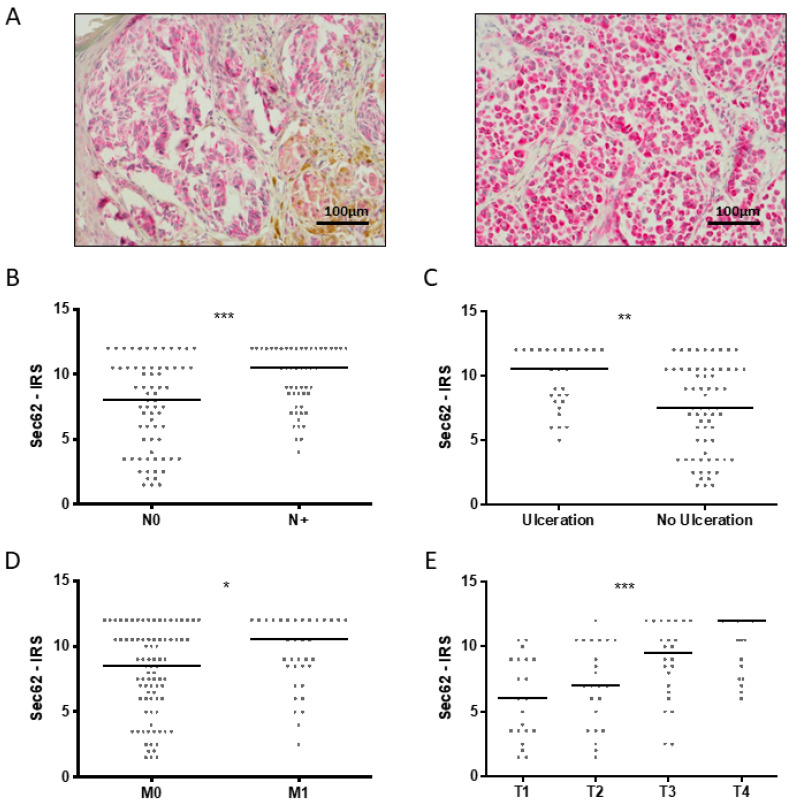
Correlation of *SEC62* expression with histopathological features. (**A**) Representative IHC-images of one Sec62-low MM (left image) and one Sec62-high MM (right image); (**B**) Sec62-immunoreactive score (IRS) for MM N0 vs. N1; (**C**) Sec62-IRS for MM with/without ulceration; (**D**) Sec62-IRS for MM M0 vs. M1; (**E**) Sec62-IRS for different T stages. In (**B**) to (**E**), every dot represents one patient, and the median is indicated by a line. Statistics: (**B**–**D**) Mann–Whitney U test; (**E**) one-way ANOVA test; *—*p* < 0.05; **—*p* < 0.005; ***—*p* < 0.001.

When correlating *SEC62* expression level (exemplary immunohistochemical stainings of one Sec62-low and one Sec62-high MM are shown in Figure 2A) with different clinical and histopathological features in melanoma, including T, N, and M stages; the presence of ulceration; sex; localization of primary tumor; and MM subtype, we found significantly higher Sec62 levels in patients with lymph node or distant metastases in comparison with those without (*p* = 0.0009 and *p* = 0.026, Figure 2B,D). Sec62 showed significantly higher expression in primary melanomas with ulceration in comparison with those without (*p* = 0.002; Figure 2C). Furthermore, *SEC62* expression showed a strong correlation with T stages, with significantly higher Sec62 levels in advanced T stages (*p* < 0.0001; Figure 2E). No significant differences in *SEC62* expression were observed depending on sex, age, melanoma subtype, and localization of the primary tumor. 

### 3.3. *SEC62* Expression in Different Melanocytic Tumors

**Figure 3:**
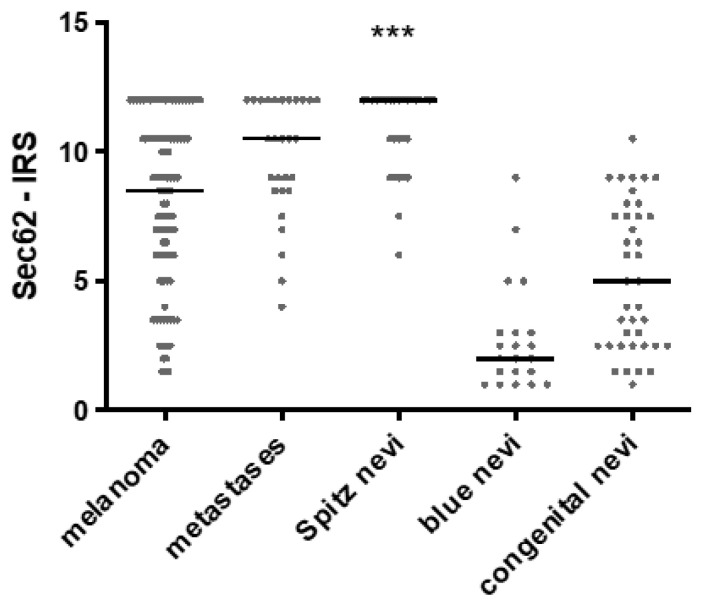
*SEC62* expression in different melanoma subtypes. Sec62-IRS delineated for primary melanoma, melanoma metastases, Spitz nevi, blue nevi, and congenital nevi. Every dot represents one patient and the median is indicated by a line. Statistics: one-way ANOVA test; ***—*p* < 0.001.

To investigate whether *SEC62* expression differed in various subtypes of melanocytic tumors, Sec62-IRS was compared between melanoma (MM; *n* = 93), melanoma metastases (MET; *n* = 28), Spitz nevi (SN; *n* = 29), blue nevi (BN; *n* = 21), and congenital nevi (CN; *n* = 38). Whereas BN and CN represent benign melanocytic tumors with no relevant risk of malignant transformation, SN are primarily benign melanocytic tumors that can progress to spitzoid melanomas and therefore are considered to be borderline melanocytic tumors. For the group of malignant melanocytic lesions, we included primary melanomas (MM) as well as distant metastases of melanomas (MET) in order to see if there are differences in *SEC62* expression between primary tumor tissue and tissue with a presumably higher metastatic potential. Highest *SEC62* expression was observed in SN, followed by MET, MM, CM, and BN (Figure 3). Multiple comparison analysis using a one-way ANOVA test showed highly significant differences in Sec62 levels all five groups (*p* < 0.0001). In detail, expression level was significantly different comparing BN and MM (*p* < 0.001), BN and MET (*p* < 0.001), BN and SN (*p* < 0.001), CN and MM (*p* < 0.001), CN and MET (*p* < 0.001), CN and SN (*p* < 0.001), and SN and MM (*p* < 0.001). No significant differences in *SEC62* expression were found between MM and MET, MET and SN, as well as BN and CN. 

### 3.4. Correlation of *SEC62* Expression with Clark Level

**Figure 4:**
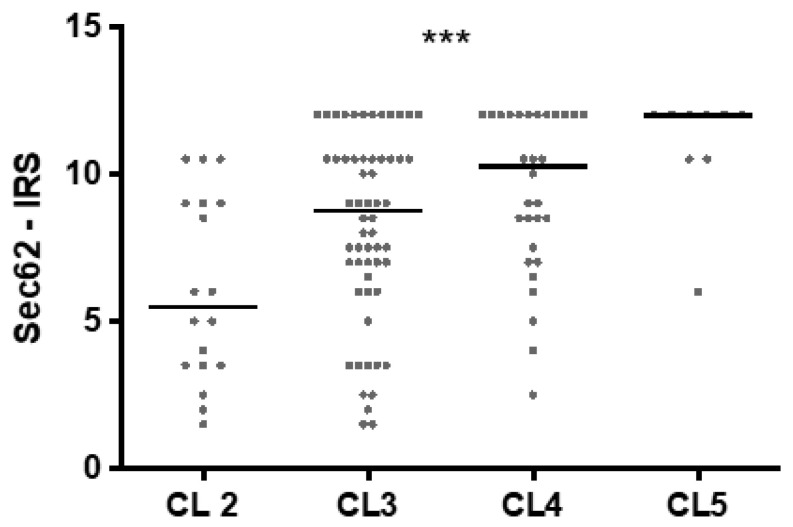
Correlation of *SEC62* expression with Clark levels (CL) in melanomas (MM + MET). Sec62-IRS delineated for Clark Levels 2 (*n* = 19) vs. 3 (*n* = 58) vs. 4 (*n* = 30) vs. 5 (*n* = 10). Every dot represents one patient and the median is indicated by a line. Statistics: one-way ANOVA test. ***—*p* < 0.001.

As Clark level is established as a relevant prognostic factor in the initial assessment of melanomas, *SEC62* expression was compared in primary melanomas with Clark levels ranging from two to five. As shown in Figure 4, *SEC62* expression showed an almost linear increase with rising Clark levels (Pearson’s r = 0.402) with median Sec62-IRS values of 5.5 (Clark level 2), 7.75 (Clark level 3), 10.5 (Clark level 4), and 12 (Clark level 5).

## 4. Discussion

For the *SEC62* gene encoded at chromosomal region 3q26.2, increasing evidence suggests a relevant role in human carcinogenesis and tumor cell biology, especially in cancer metastasis and invasiveness. An overexpression of the gene was reported for a variety of human cancers including head and neck squamous cell carcinomas (HNSCC), prostate cancer, esophageal cancer, cervical cancer, ovarian cancer, breast cancer, and non-small cell lung cancer (NSCLC) [8,12,19,20,25,26,28]. In non-small cell lung cancer, breast cancer, and head and neck cancer, high *SEC62* expression correlated with a significantly shorter overall survival, and the occurrence of lymph node and/or distant metastases [17,19,20,21]. These findings indicate a general role of *SEC62* as an oncogene in the pathogenesis of human cancer and emphasize the role of *SEC62* expression level as a clinically relevant prognostic factor in various cancer entities [22]. Only one study addressed the role of *SEC62* in skin cancer so far, which focused on 41 cases of atypical fibroxanthomas (AFX) [30]. In that study, markedly increased *SEC62* expression levels were found in the lesional tissue compared with the adjacent healthy squamous epithelium in the vast majority of cases, suggesting an oncogenic function of Sec62 in AFX. To date, no study has investigated *SEC62* expression in benign, malignant, and dysplastic melanocytic tumors, or its predictive and prognostic value, which underlines the novelty of our study and the presented data.

In concordance with the aforementioned findings, our study showed an association of high *SEC62* expression levels in melanoma with shorter OS and PFS, emphasizing the prognostic significance of Sec62 in human malignancies. The markedly elevated Sec62 levels in malignant compared to benign melanocytic tumors indicates an oncogenic function of *SEC62* in melanoma carcinogenesis. Since 3q amplifications are not a frequent finding in melanoma cells [35], the observed high Sec62 protein levels presumably are a result of specific *SEC62* gains and/or overexpression.

Regarding the functional role of Sec62 in tumor cell biology, it was shown that high Sec62 levels are able to stimulate the migration and invasion of tumor cells [8,19,28]. Additionally, high Sec62 levels help tumor cells to compensate ER stress induced by an overload of unfolded or misfolded proteins in the ER lumen using a Sec62 mediated mechanism called recovER-phagy [26]. Accordingly, we found a significant correlation of high Sec62 levels in primary melanoma with the development of lymph node and distant metastases. Hence, Sec62 seems to play a crucial role in the complex molecular mechanism of lymphatic and hematogenous metastasis in melanomas, as well, which makes Sec62 a promising target for anti-metastatic therapeutic strategies, e.g., using TFP and TG as well-characterized small molecules counteracting molecular Sec62 function [8,29]. 

Different expression levels of *SEC62* in benign and borderline melanocytic tumors showed a clear correlation with biological behavior in our study: whereas benign melanocytic lesions including CN and BN express *SEC62* at comparably low levels, borderline lesions, such as SN, which have a markedly higher risk for malignant transformation into MM, harbor significantly higher levels of Sec62. As borderline melanocytic tumors can pose significant diagnostic challenges to both clinicians and dermatopathologists, and as their clinical behavior is hard to predict, measuring *SEC62* expression levels may strengthen a diagnostic backbone for therapeutic decision-making, together with other molecular and histopathological characteristics. It is unclear why SN showed the highest *SEC62* expression levels in our study, even exceeding the levels of MM. To explain this observation, we would have to compare *SEC62* expression in SN versus spitzoid melanomas and analyze if there is again an increase in *SEC62* expression from SN to spitzoid melanoma. A direct comparison of SN with any subtype of MM has limitations considering the histological and molecular heterogeneity of malignant melanoma subtypes. One reasonable approach would be to investigate larger numbers of SN cases in order to draw robust conclusions regarding the molecular role of *SEC62* in the carcinogenesis of spitzoid melanocytic lesions. 

However, there are also some limitations to our study. As, to date, we rely on descriptive data based on immunohistochemical stainings, valid information on the potential role of *SEC62* in the tumor cell biology of melanoma cells remains elusive. In future studies, different cell culture experiments, with a focus on cell proliferation, cell migration, and cell invasion, may shed light on the detailed influence of Sec62 on relevant molecular processes of carcinogenesis, as well as the wider hallmarks of cancer cell biology. Additionally, various melanoma animal models can be used to prove if therapeutic approaches targeting Sec62, e.g., using thapsigargin and/or trifluoperazine, have the potential to (i) hamper de novo melanoma development or from precancerous lesions, (ii) suppress tumor growth, and (iii) inhibit lymphatic and hematogenous metastasis. Since almost all melanomas investigated in our study showed a strong *SEC62* expression, with IRS values between 9 and 12, the aforementioned therapeutics that have been successfully tested in *SEC62*-overexpressing head and neck cancer xenografts in vivo are promising targets for new therapeutic approaches for this difficult-to-treat cancer entity. Our study aims to provide the first valid data background on the role of *SEC62* oncogene in melanocytic tumors in order to enable functional in vitro and in vivo studies. 

## 5. Conclusions

This study is the first in the literature to investigate the role of the 3q oncogene, *SEC62,* in human melanocytic tumors and provides the first evidence for the role of Sec62 as a prognostic biomarker in melanoma. *SEC62* expression level can help to predict the clinical behavior and biological aggressiveness of borderline melanocytic lesions in particular. Hence, the analysis of Sec62 can help to guide clinical management of these patients and adds a valuable molecular tool for diagnostic workup and outcome prediction. The functional role of Sec62 in malignant melanoma, as well as its role as a potential therapeutic target, must be evaluated in further studies. 

## Figures and Tables

**Table 1:** Clinical and histopathological characteristics of melanoma patients. Sex, tumor size (T stage), involvement of lymph nodes (n stage), distant metastases (M stage), the presence of ulceration, and subtype classification are indicated for the included melanoma patients. SSM—superficial spreading melanoma, LMM—lentigo maligna melanoma, ALM—acral lentiginous melanoma, NMM—nodular melanoma.

Characteristics	Melanoma Patients *n* = 93	
	*n*	%
Sex		
male	38	40.9
female	55	59.1
Tumor size		
pT1	19	20.4
pT2	23	24.7
pT3	28	30.1
pT4	23	24.7
Lymph nodes		
pN0	66	71
pN1	27	29
Metastases		
cM0	77	82.8
cM1	16	17.2
Ulceration		
no	29	31.2
yes	64	68.8
Subtypes		
SSM	29	31.2
LMM	5	5.4
ALM	8	8.6
NMM	42	45.2

## Data Availability

Original data are available from the corresponding author upon reasonable request.

## Abbreviations

AFX	atypical fibroxanthoma
ALM	acral lentiginous melanoma
BSA	bovine serum albumin
CN	congenital nevi
ER	endoplasmic reticulum
FFPE	formalin-fixed paraffin-embedded
HNSCC	head and neck squamous cell carcinoma
IRS	immunoreactive score
LMM	lentigo maligna melanoma
MCAM	melanoma cell adhesion molecule
MET	melanoma metastases
MM	malignant melanoma
NMM	nodular malignant melanoma
n.s.	nonsignificant
NSCLC	non-small cell lung cancer
OS	overall survival
PBS	phosphate-buffered saline
PFS	progression free survival
SERCA	sarcoplasmic/endoplasmic reticulum calcium ATPase
SMM	superficial spreading malignant melanoma
SN	Spitz nevi
TFP	trifluoperazine
TG	thapsigargin
